# Communicating science-based messages on vaccines

**DOI:** 10.2471/BLT.17.021017

**Published:** 2017-10-01

## Abstract

The public health response to false information about vaccines can sometimes backfire. A series of workshops aims to help public health officials in Europe meet the challenge. Tatum Anderson reports

Italy is one of several countries in the European Region of the World Health Organization (WHO) where outbreaks of vaccine-preventable diseases have occurred in recent years. 

Since July 2016, there have been 35 measles deaths in the region: 31 in Romania, two in Italy (along with 4044 measles cases), one death in Germany and one in Portugal. 

“Recent measles outbreaks and deaths in Italy are due to the decrease in measles vaccination coverage over the last few years,” according to Dr Giovanni Rezza, Director of the Department of Infectious Diseases at Italy’s National Institute of Health.

Some parents are afraid to get their kids vaccinated because of misleading media reports, court cases related to alleged adverse reactions, scaremongering by anti-vaccination campaigners and lingering – albeit unfounded – fears about autism links, he says.

“The media often sets up a false opposition between public health officials and anti-vaccination campaigners, rather than conveying a clear message that there is an overwhelming scientific consensus in favour of nationally recommended vaccines,” Rezza says.

Part of the response to falling vaccination coverage in Italy has been the creation of a health information website called *VaccinarSì* – meaning “yes to vaccines” in 2013 by the Italian Society of Hygiene, and the health minister and other senior health officials give regular media interviews to reinforce this positive message. 

This year, the ministry introduced legislation making 10 vaccinations – polio, tetanus, diphtheria, hepatitis B, pertussis, *Haemophilus influenza* type B, measles, mumps, rubella and varicella – a school-entry requirement across Italy. Four further vaccines – for meningitis C, meningitis B, pneumonia and rotavirus – are strongly recommended by the new legislation. 

Last year, the Italian government intervened to stop *Vaxxed*, an anti-vaccination film by Andrew Wakefield, author of a fraudulent 1998 paper falsely linking the measles, mumps and rubella (MMR) vaccine to autism, from being shown in Italy.

Opposition to vaccines has long existed, ever since the first anti-vaccination leagues sprang up to oppose compulsory smallpox inoculation in the 19th century. While the arguments of vaccine opponents have not changed over time, their ability to reach large audiences with their messages has increased with the advent of the digital and social media.

One of the greatest challenges for public health authorities is how to respond to today’s proliferation of misleading information about vaccines. Studies show that simply correcting myths about vaccines may not be effective and can even backfire.

In a study of 1000 participants in the United States of America (USA) published in the journal *Vaccine* in January 2015, researchers sought to correct misperceptions that people can catch influenza from the influenza vaccine and, in a study of 1759 participants in the USA published in *Pediatrics* in April 2014, that the MMR vaccine causes autism.

When the researchers gave participants evidence-based information produced by the US Centers for Disease Control and Prevention (CDC) refuting these claims, they reduced these misperceptions, but unwittingly increased participants’ safety concerns thereby reducing their willingness to come forward or bring their children for vaccination.

Their efforts may have backfired, says Philipp Schmid from the Department of Media and Communication Science at the University of Erfurt in Germany, because repeating myths about the influenza and MMR vaccines can inadvertently reinforce them.

Avoiding the repetition of myths is one of the lessons that he and his colleagues are teaching in a series of workshops organized by WHO’s Regional Office for Europe. 

“We are not trying to change the minds of a tiny group of entrenched anti-vaccination people,” Schmid says. “These workshops prepare public health officials to communicate with a much larger group of people – mainly parents who are hesitating about whether or when to get their children vaccinated – to show them how to process the myths and messages of fear.”

“Most hesitant parents do not oppose the scientific evidence, but the appeals and messages of vaccine deniers make them feel afraid and uncertain.”

Philipp Schmid

“Most hesitant parents do not oppose the scientific evidence, but the appeals and messages of vaccine deniers make them feel afraid and uncertain,” says Schmid. “Public health authorities need to continuously build confidence and educate the public, so that people are better prepared to make vaccination decisions when they are faced with them”.

Drawing on research on the psychology of science denialism – such as the refusal to believe that climate change is happening and that HIV causes acquired immune deficiency syndrome – the workshops show participants how to deconstruct five techniques that vaccine deniers use to make their claims sound plausible.

The first is impossible expectation. For example, vaccine deniers want 100% certainty that vaccines are safe and effective. “We would never expect 100% certainty from any other product, so why should we expect it from vaccinations?”

The second is false logic. For example, some vaccine deniers falsely believe that all natural things are good and all unnatural things are bad. “In philosophy, this false logic is known as the appeal to nature,” says Schmid. “We consider life-saving surgery to be a good thing, but not natural, but when a lion kills a human it’s natural, but not good.”

The third technique is when vaccine deniers claim technical expertise, participants learn to differentiate between bogus and genuine experts. The fourth technique is to argue that governments promote vaccination because of undue influence from the pharmaceutical industry, participants learn how to counter this conspiracy theory. The fifth technique is selectivity: when arguments are built on isolated scientific papers.

After the discredited Wakefield paper was published in 1998, the proportion of children immunized with the MMR vaccine in the United Kingdom of Great Britain and Northern Ireland (UK) started falling and by 2003 had reached a low of around 80%, before improving to 90% by 2011. 

Part of the strategy to regain confidence in the MMR vaccine and other recommended vaccines in the UK was to respond to specific public concerns, says Joanne Yarwood, who was heading the immunisation information team at Health Promotion England in the early 2000s. 

Working with colleagues at the health ministry, the team ran a public information campaign and aimed to identify false information about the MMR vaccine in the UK, while doing surveys to monitor changes in public attitudes.

They found that a major source of public confusion was a multitude of conflicting media reports in local and national newspapers, and on the radio and television.

“Parents we surveyed said they wanted the facts about MMR, so we prepared leaflets and posters entitled *MMR: the facts* setting out the evidence around the vaccine,” says Yarwood, who is now national immunisation programme manager at Public Health England. 

One particular parental concern was that babies were too young to cope with a triple vaccine, so the team devised messages explaining that the MMR vaccine helps to build a baby’s immune system rather than attacking it.

“We found that consistent messaging is essential: that MMR is the safest way to protect children against measles, mumps and rubella and that the benefits of giving your child the MMR vaccine by far outweigh the risks. It took us more than 10 years to rebuild parents’ confidence in the MMR vaccine in the UK,” Yarwood says. 

WHO recommends that 95% of children should be immunized with the MMR vaccine, the herd immunity level at which all children – vaccinated and unvaccinated – are protected from measles, mumps and rubella. Since 2011, MMR immunization in the UK has been consistently above 90%, with levels exceeding those achieved before 1998.

Each country needs to find its own way to build and maintain confidence in vaccines, according to a 2014 report by a working group of WHO’s Strategic Advisory Group of Experts (SAGE) on Immunization.

The working group was tasked with developing recommendations on how countries should deal with vaccine hesitancy, which the group defined as the delayed acceptance or refusal of vaccines despite their availability. 

The group found that religious beliefs, lack of trust in health authorities and providers, safety concerns and a lack of perceived benefit of vaccines were among the reasons for vaccine hesitancy around the world.

For Robb Butler, programme manager of vaccine preventable diseases and immunization in WHO’s Regional Office for Europe, transparency and education are key. 

“Public health authorities need to brief the media regularly on these issues to ensure more balanced and well-informed reporting during outbreaks and vaccine safety scares, and to ensure an open line for questions when they arise,” Butler says, adding that the public also needs to be educated about immunization and that this should start at school. 

“Efforts to build public confidence in vaccination and make the public more resilient to false information need to be ongoing and not just in times of crisis, otherwise we are firefighting,” Butler says. 

Heidi J. Larson, a member of the SAGE working group who heads the Vaccine Confidence Project at the London School of Hygiene and Tropical Medicine, says it’s also essential to challenge misinformation and rumours as soon as they surface.

“As a public health community we are now in a difficult situation because we weren’t paying enough attention to public concerns in the past,” says Larson. 

“We've invested in more and more vaccines and much less in bringing the public along with us. We need to pay attention to the public and listen to their concerns much earlier on,” Larson says.

**Figure:**
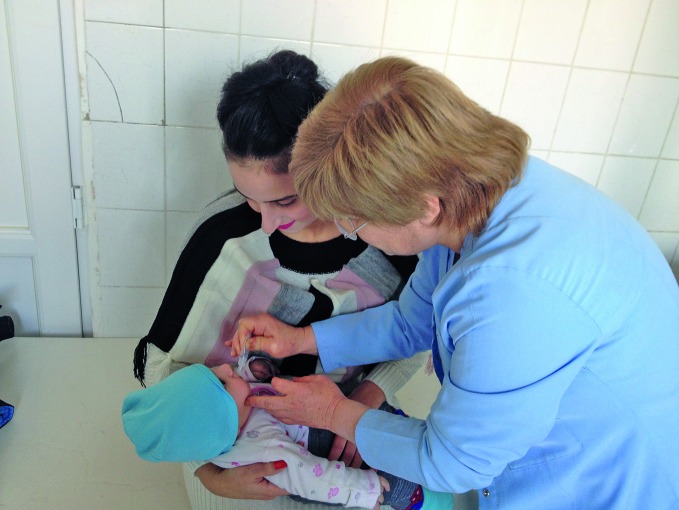
A child receives the polio vaccine in Armenia

WHO

